# Fault Diagnosis by Multisensor Data: A Data-Driven Approach Based on Spectral Clustering and Pairwise Constraints

**DOI:** 10.3390/s20247065

**Published:** 2020-12-10

**Authors:** Massimo Pacella, Gabriele Papadia

**Affiliations:** Department of Engineering for Innovation, University of Salento, 73100 Lecce, Italy; gabriele.papadia@unisalento.it

**Keywords:** semi-supervised classification, spectral clustering, PCA, fault detection, fuel-injection system

## Abstract

This paper deals with clustering based on feature selection of multisensor data in high-dimensional space. Spectral clustering algorithms are efficient tools in signal processing for grouping datasets sampled by multisensor systems for fault diagnosis. The effectiveness of spectral clustering stems from constructing an embedding space based on an affinity matrix. This matrix shows the pairwise similarity of the data points. Clustering is then obtained by determining the spectral decomposition of the Laplacian graph. In the manufacturing field, clustering is an essential strategy for fault diagnosis. In this study, an enhanced spectral clustering approach is presented, which is augmented with pairwise constraints, and that results in efficient identification of fault scenarios. The effectiveness of the proposed approach is described using a real case study about a diesel injection control system for fault detection.

## 1. Introduction

**Figure 1:**
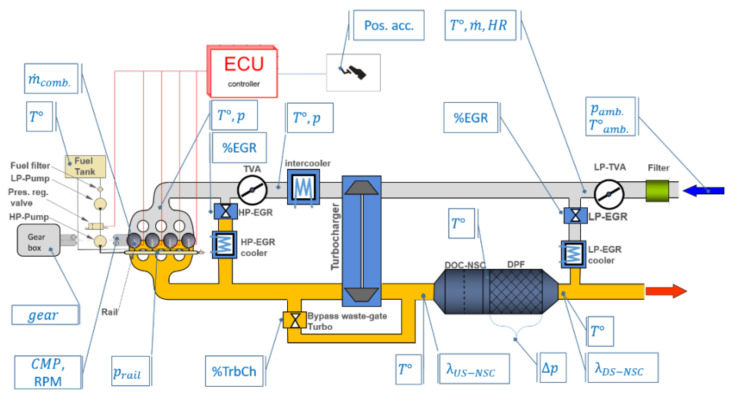
Layout schema of an internal combustion engine [1,2].

To accurately inspect the operating conditions of an internal combustion engine, several sensors are used to collect real-time measurements. For instance, to control fuel consumption and emissions of pollutants into the environment, the exhaust after-treatment process of an internal combustion engine is monitored by various classes of sensors. In [1,2] is discussed the problem of fault diagnosis based on data sampled by a large number of sensors, measuring, for example, the vehicle velocity, the average engine rotational speed, and the air mass flow. The measurements sampled by the sensors, disseminated on the supply and after-treatment line of the engine (Figure 1), contain a high amount of information, which is fundamental not only in the regulation of the systems but also to provide an interpretative model of the process, which facilitates a rapid diagnosis of potential faults.

**Figure 2:**
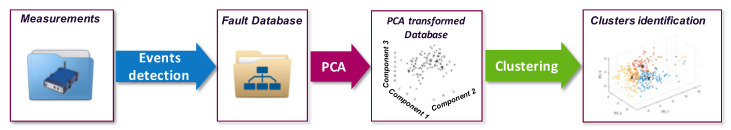
A frame of fault diagnosis based on multisensor data.

Considering measurements are collected faster than they are analyzed, automatic fault diagnosis procedures are required to rapidly and efficiently process data and provide detailed results [3]. A frame of fault diagnosis based on multiple sensors includes: (i) data acquisition; (ii) feature extraction; (iii) fault diagnosis. In the signal acquisition step, many types of sensors are considered, which provide a large number of signals. In the second step, feature extraction aims to extract representative features from the collected signals through dimension reduction. The objective is to separate sensitive from insensitive information that may affect the diagnosis results as well as computational efficiency. In fault diagnosis, clustering is used for determining groupings within data (with higher similarity within groups and lower similarity among groups) and assigning labels to data points according to these groupings. In Figure 2, the flow of a conventional approach is represented, in which the first step allows the definition of data-filtering criteria for events detection. Once an organized database of elements is obtained, it is possible to apply a feature extraction approach (such as the Principal Component Analysis—PCA), which returns a transformed database. Clustering is implemented to separate data into clusters (families of events), which allow experienced personnel to diagnose the fault. The approach schematized in Figure 2 was exploited in [1]. A similar approach is implemented in this study.

Several studies presented in the literature for fault diagnosis are based on classification methods (support vector machine, naive Bayes, and logistic regression). These approaches are basically supervised because labeled training data with known fault classes are employed to train the classifier first. Subsequently, the classifier processes new data to diagnose potential faults by matching the patterns against the measurement data [4]. However, reliable measurements under a specific label (known fault condition) may not be available in actual applications. When labeled data are not available for training, the unsupervised classification of measurement data provided by several sensors during a fault event should be considered to support fault diagnosis. The unsupervised classification is based on partitional clustering of profile data to isolate the fault events in a restricted number of scenarios, each one described by a reference pattern. Then, this pattern could be examined by an expert for decision-making, in other words, to find root causes.

In this paper, a semi-supervised data-driven approach is discussed, in which combined labeled and unlabeled measurement data are used to train the model. Our proposed approach is based on a clustering method in which we assume to have information about pairs of vectors that do not belong to the same cluster (cannot-links) and information about pairs of vectors that belong to the same cluster (must-links) [5]. This information, which may be available from experienced personnel concerning a small subsample of data measurements, may lead to enhanced performance in the clustering process of data.

The most common clustering technique is the *K*-means and its variants [6,7]. These methods partition the data into several *K* groups with the goal is to minimize a within-cluster dispersion measure. However, the *K*-means algorithm performs poorly in case the dataset is not the union of well-separated spherical structures. On the other hand, spectral methods [8,9] are recommended to handle irregularly shaped clusters by using the information of an affinity matrix, which is used for measuring the similarity among data points. Spectral clustering (SC) is an important subject of research in recent years [9]. If the shape of clusters deviates from well-separated spherical structures [10], for which *K*-means performs well [11], SC is an effective approach. The SC method has been shown robust concerning the geometry of the clusters, noise, and outliers [12].

The SC approach reduces clustering to a problem of graph partitioning [13,14,15,16]. The first step of SC involves forming a positive semi-definite affinity matrix with each entry that refers to the measure of similarity linking each pair of data points. Then, by consulting only a few eigenvalues and eigenvectors of such a matrix, SC maps the data points to $R^{K}$ (where *K* is the number of selected eigenvectors of the matrix). This mapping involves the projection of data onto a new space, in which points form tight clusters, and simple clustering methods can be used. SC algorithms are particularly well suited for clustering in a high-dimensional setting. Such is the case of signals acquired by a multisensor monitoring system as emerged with present control systems, which govern the functioning of an internal combustion engine. One of the most significant issues in this application refers to the high number of variables that define the state of the modeled process. To increase clustering accuracy and to reduce the computational cost, it is necessary to reduce the dispersion of raw data to allow a meaningful classification.

The SC approach is analyzed in this study for multisensor data. The effectiveness of the SC approach is illustrated using a real case study concerning a diesel injection control system for fault detection. In a diesel engine, fuel injection into the cylinders is possible thanks to reliable injectors [17]. Nevertheless, a fault may occur due to a flash opening of the injector, which causes an unspecified pressure drop in the fuel rail. The diagnostic system interprets these events as the repeated opening of a worn-out relief valve. This safety component is equipped on a heavy-duty diesel engine to prevent high pressures in the fuel rail, letting fuel to flow back, and so avoiding the system to move into a dangerous condition [18]. In our case study, the diagnosis of the fault cannot be efficiently performed by practitioners due to a large number of sensors, and hence data-driven approaches are required to support the root-cause analysis of a fault.

The contributions of our research are as follows: (i) we propose a new approach for including pairwise constraints information into SC; (ii) we prove that this approach improves the quality of classification using a real case study about a diesel injection control system for fault detection. Despite the specific case study described in this paper, the proposed approach can be exploited in several applications, in which multisensor measurement data are collected on a process. In particular, in the manufacturing field, where the final quality of the manufactured part is more and more often related to the faults of the machining processes [19]. Moreover, the proposed methodology may have a widespread application in other experimental settings of fault diagnosis of interest in the recent literature, such as vibrational signals of induction motors [20] or bearing faults in rotational machinery [21,22].

The outline of the present paper is as follows. Section 2 provides an overview of feature extraction for process data. Section 3 presents an overview of *K*-means clustering methods while SC and the proposed SC augmented with pairwise constrain information is described in subsequent Section 4. The effectiveness of the clustering operation is measured by the validation indices described in Section 5. Numerical validation of representative datasets is presented in Section 6, where the case study concerning an injection control system for a diesel engine is considered. The different performances of *K*-means and SC are compared in demonstrating the fault scenario. Finally, Section 7 provides conclusions of this study and presents directions for future research.

## 2. Feature Extraction of Process Data with a High Number of Variables

A large number of sensors employed to monitor the state of the process may result in a challenge for a study aimed to define a data-driven approach for fault diagnosis. PCA is a well-known method to reduce the dispersion of the multisensor measurements and their dimensionality. PCA results in the transformation of original variables into a small number of features (principal components, PCs).

The preliminary operation of the PCA approach consists of computation of the sample covariance matrix and its eigendecomposition. The resulting eigenvalues are sorted in decreasing order, where each eigenvalue is related to the fraction of variance explained by the linked PC. Corresponding orthogonal eigenvectors describe a basis of space whose directions are referred to as the maximum variability of the data. The advantage of PCA as a dimensionality reduction algorithm consists of reducing the number of variables while preserving as much variability as possible of the initial raw measurements. In [1], the PCA is used as a feature extraction method for the clustering of multichannel profiles, where analysis concerns the root causes of the fault related to an emission control system in a diesel engine.

Consider the case of a *P*-sensor data, of *M* samples. A generic sample is stored in a matrix designated as $X\in R^{P\times M}$ and addressed by indexes: *j*, *i* related to rows and columns of X, respectively. Let $\bar{x}=\frac{1}{M}\sum_{i=1}^{M}x_{i}$ be the average vector of data and let $x_{i}^{c}=x_{i}-\bar{x}$ be the centered vector obtained from $x_{j}$ by subtracting the average vector. The entire dataset can be represented by matrix $X^{c}\in R^{P\times M}$ (the rows represent the *P* variables and the columns are the *M* samples $x_{i}^{c}$).

The aim of PCA is to solve the problem of approximating the data matrix $X^{c}$ with another matrix ${\tilde{X}}^{c}$ which has a lower rank, where the approximation objective is to minimize the distance between $X^{c}$ and ${\tilde{X}}^{c}$. *N* is a given upper bound for the rank of matrix ${\tilde{X}}^{c}$ (N<P). Hence, denoting by $\tilde{U}$ the matrix formed by the first *N* columns of U, which correspond to the first *N* larger singular values of $X^{c}$, a data sample vector of *P* points $x_{i}$ (i=1,…,M) is projected to a feature space as ${\tilde{U}}^{T}\left(x_{i}-\bar{x}\right)$. This is the vector of *N* coordinates $t_{i}=\left(t_{i1},\ldots ,t_{iN}\right)$ which represent the so-called *scores* (PC-features) of vector $x_{i}$. Let $T={\left\{t_{i}\right\}}_{i=1}^{M}$ represent the dataset of scores resulting from PCA.

## 3. *K*-Means Clustering

The *K*-means algorithm is a universal technique in clustering due to its simplicity and ease of use, despite it suffers from setting initial conditions and a non-spherical-shape characteristic of the dataset. Let $T={\left\{t_{i}\right\}}_{i=1}^{M}$ represent the original dataset. A generic point $t_{i}\in R^{N}$, assigned to a group, has high intra-cluster similarity (SSW) with the remaining points belonging to the same cluster while it has low inter-cluster similarity (SSB) with the remaining points assigned to different groups.

These parameters are analytically expressed as $SSW=\sum_{k=1}^{K}N_{k}\sum_{t_{i}\in C_{k}}d\left(t_{i},c_{k}\right)$ and $SSB=\sum_{k=1}^{K}N_{k}\cdot d\left(c_{k},\bar{c}\right)$. Where $d:R^{N}\times R^{N}\to \left[0,\infty \right)$ is a distance metric in $R^{N}$ (in this work, the Euclidean distance is used). SSW represents the sum of the squared distance between each *i*-th data point $t_{i}$ and its closed centroid: $c_{k}=\frac{1}{|C_{k}|}\sum_{t\in C_{k}}t$, in other words the barycentre of the *k*-th cluster $C_{k}$, where $N_{k}$ is the number of points of the *k*-th cluster. SSW represents the within-cluster variance. The objective of clustering is to find cluster centroids that minimize SSW (tight clusters). SSB is the sum of the squared distance between $c_{k}$, previously introduced, and $\bar{c}$, which is the mean position of all *K* centroids. SSB represents the between-cluster variance. Clustering should maximize SSB (clusters well separated).

To solve such a problem, minimize SSW and maximize SSB, the *K*-means algorithm [23] executes two main steps: (i) initialization of *K* centroids uniformly distributed between points to be classified; (ii) consequent aggregation of points around centroids, using distance as a criterion of similarity. Once a cluster of points has been settled, the centroid is determined as a weighted average of the points. This step, repeated for each cluster, is followed by a re-calculation of the clusters and related centroids. Iterating the process, when the position of the centroids does not vary significantly, the algorithm reaches convergence.

In [24], several conditions were considered under which the original *K*-means algorithm fails or requires a long time before it converges to an adequate solution. As a result, a variant of the original algorithm, named *K*-means++, was introduced. This variant produces a better classification along with a reduction of the SSW parameter and thus compactness of the clusters compared to the initial *K*-means.

In particular, in *K*-means++, a specific way of choosing centers for the *K*-means algorithm is implemented. Let $d(t^{\prime })$ denote the shortest distance from a data point to the closest center chosen. The first centroid is arbitrarily chosen in the overall set of points to be grouped. Remaining K−1, are chosen according to the probability distribution $\frac{d{\left(t^{\prime }\right)}^{2}}{\sum_{t\in T}d{\left(t\right)}^{2}}$. Once the *K* centroids are labeled, the *K*-means++ algorithm proceeds as the original *K*-means algorithm. When compared to the original *K*-means algorithm, the *K*-means++ shows a better classification accuracy and a faster convergence. We summarize the pseudocode implemented in this study in Algorithm 1.
**Algorithm 1***K*-means ++ clustering with distance metric *d* (use Euclidean distance)**Input:**$T={\left\{t_{i}\right\}}_{i=1}^{M}$ (Data), $K\in N_{0}$**Output:***Y* (Labels)1:Select first centroid $c_{1}$ uniformly at random from ${\left\{t_{i}\right\}}_{i=1}^{M}$.2:Select a new centroid $c_{i}$ as $c_{i}=t^{\prime }\in T$ with probability $\frac{d{\left(t^{\prime }\right)}^{2}}{\sum_{t\in T}d{\left(t\right)}^{2}}$; d(t) denotes the shortest distance from generic point t to the nearest centroid already selected.3:Repeat step 2 until *K* centroids have been collected: $C=\{c_{1},c_{2},\ldots ,c_{K}\}$.4:For each i∈{1,…,K}, set the cluster $C_{i}$ to be a group of points in T that are the nearest to $c_{i}$ then they are to $c_{j}$ for j≠i.5:For each i∈{1,…,K}, set $c_{i}=\frac{1}{|C_{i}|}\sum_{t\in C_{i}}t$, in other words the barycentre of $C_{i}$.6:Loop steps 4 and 5 until set C no longer changes.7:Compute *Y* as an labels array containing cluster indexes of each ${\left\{t_{i}\right\}}_{i=1}^{M}$.

### Selecting the Number of Clusters by Elbow Method

The *K*-means algorithm requires the preliminary information concerning the number of clusters and so the number of centroids around which to aggregate the nearest points. This feature makes the *K*-means algorithm particularly attractive in unsupervised classification problems. Since the data structure is not known, it is convenient to use a single degree of freedom consisting of the number of clusters *K*.

The compactness of clusters is one of the criteria used to assess the quality of the clustering. This characteristic is quantified by parameter SSW. Selecting the optimal number of clusters is usually based on SSW(K) as a function of variable $K\in N_{0}$. This function is decreasingly monotonous since as more centroids are introduced, smaller are the clusters, and consequently, the smaller is SSW(K) (compactness criterion). There is an optimal value of *K* above, in which the SSW(K) parameter does not decrease appreciably. This condition represents an elbow of the curve followed by a plateau for increasing *K*-values. Search for optimal *K* results in the identification of the maximum curvature point of SSW(K). In [25], an algorithm called *Kneedle* is provided to determine the maximum curvature point in a discrete distribution. This approach allows optimization by calibrating appropriate threshold values that influence the sensitivity of the technique to converge to the optimum point. In the present study, *Kneedle* is used on offline data resulting from the experiments. The algorithm is implemented following the *K*-means to calibrate, in a closed-loop, the *K* input for clustering iteration.

## 4. Spectral Clustering

The *K*-means performs well if data fit a Gaussian model. On the other hand, SC does not pre-assume any model. SC aims to optimize certain criterion that measures the quality of graph partitions. Dissimilarly from the *K*-means algorithm that works directly on data points, the SC method starts from an affinity matrix that measures the pairwise similarity of the data points. This corresponds to a graph partition, such that the intra-group edge weights are high and the inter-group edge weights are low. Mathematically, this is the problem of finding eigenvectors of the Laplacian graph from the affinity matrix and then clustering eigenvectors into clusters.

Given the dataset $T={\left\{t_{i}\right\}}_{i=1}^{M}$, whereby there is some degree of similarity between two generic $t_{i}$ and $t_{j}$ points. Consequently, it is possible to create a graph that reflects the properties of T, which can be efficiently processed by the clustering algorithm. The first step of SC involves forming a positive semi-definite *affinity* matrix $A\in R^{M\times M}$ such that each entry $a_{ij}$ represents the affinity between data $t_{i}$ and $t_{j}$. Standard SC methods first construct a graph G=(T,A), where T denotes the set of vertices and $a_{ij}$ gives the weight of the edge that connects $t_{i}$ and $t_{j}$. In these terms, the clustering objective is reduced to identifying that particular partition (a subset of vertexes) of the graph whose edge weights show low values with contiguous partitions. The connections between internal vertexes are associated with high similarity indexes. The partitioning of the graph is then obtained by assigning large edge weights within each cluster and small edge weights between each cluster.

The original formulation of SC uses the traditional Gaussian kernel-based similarity. Let $d:R^{N}\times R^{N}\to \left[0,\infty \right)$ be a distance metric in $R^{N}$ (in this work, the Euclidean distance is used). Set $a_{ij}=\exp\left(-\frac{d{\left(t_{i},t_{j}\right)}^{2}}{2\sigma^{2}}\right)$, if i≠j, 0 otherwise. σ is a global scaling parameter for every object pair, which always has to be set manually. However, the effect of σ is important, and the optimal σ for different data turned out to be very different. Moreover, there may not be a single value of σ that works well for all the data. In fact, when σ is set small, $a_{ij}$ cannot effectively capture the high correlation between distant objects in a large sparse cluster. On the contrary, when σ is set large, objects from different but nearby dense clusters will then more likely to be misjudged as similar.

To address the issue, a local scale parameter for each point allows self-tuning of the point-to-point distances according to the local statistics of the neighborhoods surrounding points $t_{i}$ and $t_{j}$. The local scaling parameter $\sigma_{i}$ for each object $t_{i}$ is defined as the distance between $t_{i}$ and its *l*-th nearest neighbor (*l* can be empirically set). For an object $t_{i}$ in a sparse cluster, $\sigma_{i}$ is large. This enlarges the similarity between $t_{i}$ and other distant objects in the same cluster of $t_{i}$. Also, a dense cluster gives a small $\sigma_{i}$, which effectively decreases the similarity between $t_{i}$ and objects from nearby clusters. Thus, the affinity between the points $t_{i}$ and $t_{j}$ can be written as: $a_{ij}=\exp\left(-\frac{d{\left(t_{i},t_{j}\right)}^{2}}{\sigma_{i}\sigma_{j}}\right)$.

Scale parameters $\sigma_{i}$ calibrate the similarity index according to the dispersion of points around generic $t_{i}$. For each index *i*, $\sigma_{i}$ was computed as a self-calibrating parameter based on point distribution [26]. The selection of the local scale $\sigma_{i}$ can be done by considering the local statistics of the neighborhood of point $t_{i}$. A simple approach, which is used for the experiments in this paper, is: $\sigma_{i}=d\left(t_{i},t_{knn}\right)$, where $t_{knn}$ is the *k* nearest neighbor of point $t_{i}$ [27].

In our experiments, we employed the value of knn=7, which showed good results even for high-dimensional data. However, compared to methods that use a global scale, this approach comes with a slightly higher computational cost considering it calls for a knn search for each data point in the process of forming the affinity matrix. Other approaches discussed in the literature for defining the affinity matrix are the Dominant Neighborhoods [28] and the Consensus of knn [29].

### 4.1. Laplacian Graph

Let $A\in R^{M\times M}$ with the (i,j) entry $a_{ij}=\exp\left(-\frac{d{\left(t_{i},t_{j}\right)}^{2}}{\sigma_{i}\sigma_{j}}\right)$ be the affinity matrix. The degree matrix D is a diagonal matrix associated with A with $d_{ii}=\sum_{j=1}^{M}a_{ij}$ be the sum of A’s *i*-th row. The combinatorial, degree-normalized and symmetric normalized Laplacian graph are defined as follows.

(1) $$L=D-A\;,\;L_{\mathrm{norm}}=D^{-1}L\;,\;L_{\mathrm{sym}}=D^{-\frac{1}{2}}LD^{-\frac{1}{2}}$$

Different types of normalization can be considered for SC. For example, the *normalized cuts* (NCuts) [30] method employs random walk-based normalization $L_{\mathrm{norm}}=D^{-1}\left(D-A\right)=I-D^{-1}A$ while the *Ng-Jordan-Weiss* (NJW) [8] method uses symmetric normalization $D^{-\frac{1}{2}}\left(D-A\right)D^{-\frac{1}{2}}=I-D^{-\frac{1}{2}}AD^{-\frac{1}{2}}$, where I is the identity sparse matrix. In this study, the normalized SC is implemented as it maximizes within-cluster similarity and minimizes between-cluster similarity, while unnormalized SC only minimizes between-cluster similarity [9]. The matrix L is semi-positive definite and its eigenvalues are in the interval [0,2]. The eigenvalues of $D^{-\frac{1}{2}}AD^{-\frac{1}{2}}$ range in the interval [−1,1].

Therefore, SC with $L_{\mathrm{sym}}$ combined to NJW algorithm [8] is implemented. We denote the eigenvalues of $L_{\mathrm{sym}}$ (identical to those of $L_{\mathrm{norm}}$) by $\lambda_{1}\leq \ldots \leq \lambda_{n}$, and the corresponding eigenvectors by $\phi_{1},\ldots ,\phi_{n}$. To cluster the data into *K* groups according to [8], the first step consists of computing an M×K matrix Φ whose columns are given by ${\left\{\phi_{j}\right\}}_{j=1}^{K}$. The rows of Φ are then normalized to obtain the matrix V, that is $v_{ij}=\Phi_{ij}/{\left(\sum_{j}\Phi_{ij}^{2}\right)}^{1/2}$. Let ${\left\{v_{i}\right\}}_{i=1}^{M}\in R^{K}$ denote the rows of V. Eventually, *K*-means is applied to cluster the ${\left\{v_{i}\right\}}_{i=1}^{M}$ into *K* groups, which defines a partition of data ${\left\{t_{i}\right\}}_{i=1}^{M}$. $L_{\mathrm{norm}}$ can be used instead [30].

Choosing *K* is a significant aspect of the SC method, and in fact, various approaches have been proposed in the literature [31,32]. The eigenvalues of $L_{\mathrm{sym}}$ can be used to estimate the number of clusters by considering the largest empirical eigengap $\hat{K}={arg max}_{i}\lambda_{i+1}-\lambda_{i},$. This heuristic estimate is called the *eigengap* statistic [9].

Basically, we use multiple eigenvectors to embed each data point into a low-dimensional space to preserve the significant difference in normalized similarity. Then, the *K*-means algorithm can be used to group the points in the embedding space. *K*-means is applied to cluster the ${\left\{v_{i}\right\}}_{i=1}^{M}\in R^{K}$ into *K* groups.

The SC approach reflects the key objective of non-hierarchical clustering, consisting of clusters of points with high similarity (intra-clusters) and low similarity between points belonging to different clusters (inter-clusters). In the *K*-means algorithm, this target is reached through iterative optimization. SC, using the graph representation, learns the structure of the set of points intrinsically [9]. The choice of *K* in SC has been analyzed by many authors in the literature [26,31,32]. We summarize the pseudocode implemented in this study in Algorithm 2.
**Algorithm 2** Spectral Clustering with metric *d* (Euclidean distance)**Input:**$T={\left\{t_{i}\right\}}_{i=1}^{M}$ (Data)**Output:***Y* (Labels)1:Select the local scale $\sigma_{i}=d\left(t_{i},t_{knn}\right)$ where $t_{knn}$ is the 7th nearest neighbor of point $t_{i}$.2:Compute the affinity matrix $A\in R^{M\times M}$ with $a_{ij}=\exp\left(-\frac{d{\left(t_{i},t_{j}\right)}^{2}}{\sigma_{i}\sigma_{j}}\right)$.3:Compute the diagonal degree matrix $D\in R^{M\times M}$ with $d_{ii}=\sum_{j=1}^{M}a_{ij}$.4:Form the symmetric normalized Laplacian graph $L_{\mathrm{sym}}=I-D^{-\frac{1}{2}}AD^{-\frac{1}{2}}$.5:Compute the eigendecomposition ${\left\{\left(\phi_{i},\lambda_{i}\right)\right\}}_{i=1}^{M}$, sorted so that $\lambda_{1}\geq \lambda_{2}\geq \ldots \geq \lambda_{M}$.6:Estimate the number of clusters *K* as $\hat{K}={arg max}_{k}\lambda_{k+1}-\lambda_{k}$.7:For 1≤i≤M, let $v_{i}=\left(\phi_{1}\left(t_{i}\right),\phi_{2}\left(t_{i}\right),\ldots ,\phi_{K}\left(t_{i}\right)\right)/\left|\right|\left(\phi_{1}\left(t_{i}\right),\phi_{2}\left(t_{i}\right),\ldots ,\phi_{K}\left(t_{i}\right)\right){\left|\right|}_{2}$ define the (row normalized) spectral embedding.8:Compute labels *Y* by running *K*-means on the data ${\left\{v_{i}\right\}}_{i=1}^{M}$ using $\hat{K}$ as the number of clusters.

### 4.2. Spectral Clustering Variants

In this study, we implemented a standard SC method, the NJW. It is worth notice that different SC methods emerged in the literature, which can be classified into the following three categories.

- **Power iteration (PI)-based methods**: PIC (Power Iteration Clustering) [33], DPIC (Deflation-based Power IterationClustering) [34], and DPIE (Diverse Power Iteration Embedding) [34] apply PI (Power Iteration) to generate pseudo-eigenvectors as a replacement of eigenvectors.
- **Multi-scale-data-oriented methods**: ZP and FUSE. The first [26] is a self-tuning SC method. It uses eigenvector rotation to estimate the number of clusters. The second [35] is an SC method based on power iteration and Independent Component Analysis.
- **Matrix-reconstruction methods**: This group of methods constructs a new coefficient matrix based on which new similarity matrix as a replacement of the original one. The main representative of the SC algorithm in this group is ROSC (Robust spectral clustering on multi-scale data) [36], which generates a matrix with a grouping effect.

### 4.3. Pairwise Constraints in Spectral Clustering

The basic idea of an SC method is to obtain the graph partition by the eigendecomposition of Laplacian graph [14]. The algorithm searches the space of possible organizations of the data, preferring those which group similar instances together and keep dissimilar instances apart. Defining pairwise similarity for an effective SC method is fundamentally challenging, given complex data that are often of high dimension and heterogeneous nature.

Moreover, an SC method is based on matching, and it can easily use the pairwise constraint information provided by practitioners. In most cases, experienced personnel have some prior or background knowledge. How to use prior or background knowledge to improve the cluster quality and promote the efficiency of clustering data has become a research topic in recent years. In this study, we aim to insert supplementary pairwise similarity between samples in the original SC algorithm. The goal is to construct more meaningful affinity graphs for enhanced SC.

Two types of pairwise constraints are considered. The must-link constraints show that two sample points should be embedded in the same cluster. The cannot-link constraints show that two sample points should be divided into different classes. The number of distinct constraints ranges from 1 to $\frac{1}{2}M\left(M-1\right)$, since constraints are by definition symmetric.

In our study, we considered SC aided by the addition of constraints, which serve to restrict the search space and to guide the search through it. We implemented both must-link and cannot-link constraints. The former constraint specifies that two data instances have to be in the same cluster; the latter constraint specifies that two data instances must not be placed in the same cluster.

Let the relation of must-link constraints (two points have to be in the same cluster) be defined as $\mathrm{ML}=\{\left(t_{i},t_{j}\right)\}$, and the relation of cannot-link constraints (prevent two points being from the same cluster) as $\mathrm{CL}=\{\left(t_{i},t_{j}\right)\}$. Thus, the affinity matrix is modified as follows.

(2) $$a_{ij}=\left\{\begin{array}{cc} 1, & \mathrm{if}\;i\neq j\;\mathrm{and}\;(t_{i},t_{j})\in \mathrm{ML};\\ 0, & \mathrm{if}\;i=j\;\mathrm{or}\;(t_{i},t_{j})\in \mathrm{CL};\\ \exp\left(-\frac{d{\left(t_{i},t_{j}\right)}^{2}}{\sigma_{i}\sigma_{j}}\right), & \mathrm{otherwise}. \end{array}\right.$$

## 5. Internal Clustering Validation Measures

In [37], a comprehensive study of 11 internal validation measures was presented by evaluating their performance on a known dataset. In our study, Caliński-Harabasz (CH), Davies-Bouldin (DB), and Silhouette (*S*) are the internal validation measures used.

The CH index evaluates the cluster validity based on the average between- and within-cluster variance. It can be defined as follows.

(3) $$CH=\frac{M-K}{K-1}\cdot \frac{SSB}{SSW}$$

where *M* is the total number of elements, and *K* is the number of clusters chosen in the classification. SSB and SSW represent inter-cluster and intra-cluster dispersion. A greater CH index shows a better clustering result.

The DB index can be defined as follows.

(4) $$DB=\frac{1}{K}\sum_{k=1}^{K}\max_{k^{\prime }\neq k}\frac{\left({\bar{d}}_{k}+{\bar{d}}_{k^{\prime }}\right)}{d_{k,k^{\prime }}}$$

where *K* is the number of clusters, ${\bar{d}}_{k}$ is the mean distance between the elements of the *k*-th cluster and their respective centroid, similarly for ${\bar{d}}_{k^{\prime }}$. $d_{k,k^{\prime }}$ represents the distance between the centroid of the *k*-th and the $k^{\prime }$-th cluster. According to the criteria of compactness and separation, the DB parameter must be as small as possible.

Another validation index, which quantifies the compactness and separation between clusters, is *S* (*Silhouette*) index. Let function $s(t_{i})$ be defined as follows:

(5) $$s\left(t_{i}\right)=\frac{b\left(t_{i}\right)-a\left(t_{i}\right)}{\max\left(a\left(t_{i}\right),\;b\left(t_{i}\right)\right)},$$

where $a(t_{i})$ represents the mean distance between the generic point $t_{i}$ and the remaining points assigned to the same cluster; $a(t_{i})$ measures compactness. The $b(t_{i})$ element is the smallest mean distance between point $t_{i}$ and residual points assigned to the remaining clusters; $b(t_{i})$ is an index of separation between clusters. Parameter $s(t_{i})$ is representative of how much a point $t_{i}$ belongs to the assigned cluster. From the definition of $s(t_{i})$, valid for the single point, it is possible to define the global *Silhouette* index *S*:

(6) $$S=\frac{1}{K}\sum_{k=1}^{K}\frac{1}{N_{k}}\sum_{t_{i}\in C_{k}}s\left(t_{i}\right)$$

where *K* is the number of clusters, $N_{k}$ is the number of elements assigned to the *k*-th cluster. Higher is *S* (at most tending to 1), better is the corresponding clustering solution.

## 6. Fault Diagnosis of an Injection Control System

In the development of a modern diesel engine, numerous technologies are employed to reduce fuel consumption and the emission of pollutants into the environment. Two examples are the selective catalytic reduction system and the high-pressure common-rail (HPCR) system. In particular, the HPCR is a fuel-injection system equipped with a storage chamber, in which fuel is stored under pressure, and a rail pipe, which provides fuel to the injectors. By the HPRC, the Engine Control Unit (ECU) regulates and optimizes the combustion process in a very accurate manner.

The adequate operation of the HPRC is guaranteed by electronically controlling most of its subcomponents through triggers modulated by the ECU. These signals are the result of control logic obtained by comparing measurements recorded by sensors and calibrated thresholds. By using electronic regulation, the injection pressure can be adjusted according to both the rotational speed of the engine and the torque demands of the driver (through the accelerator pedal).

Fuel injection into a cylinder is possible thanks to accurate injectors [17]. Nozzle opening occurs indirectly by perturbing the balance of hydraulic forces upstream of the needle. Using a high-pressure gradient, the energizing of a solenoid valve allows refueling of the fuel through calibrated holes, resulting in a dragging effect by lifting the needle. When the energizing of the solenoid valve coil stops, the hydraulic state is restored. Next, the initial equilibrium of forces along the injector valve rod is re-established. The result coincides with the needle falling and the nozzle closing.

**Figure 3:**
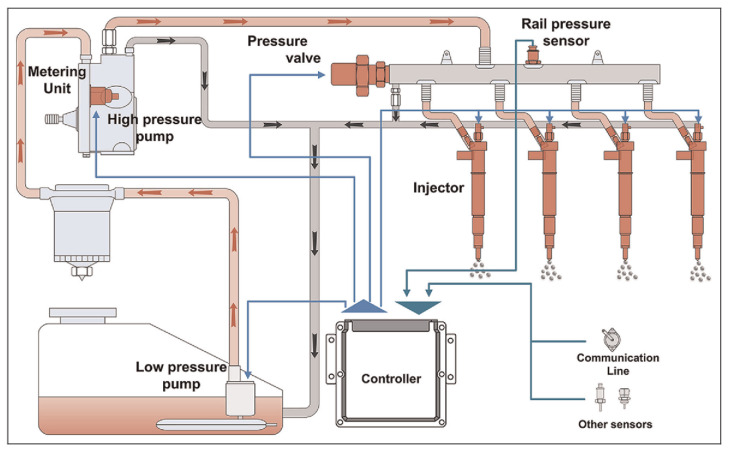
Layout schema of high-pressure common-rail (HPCR) system.

**Figure 4:**
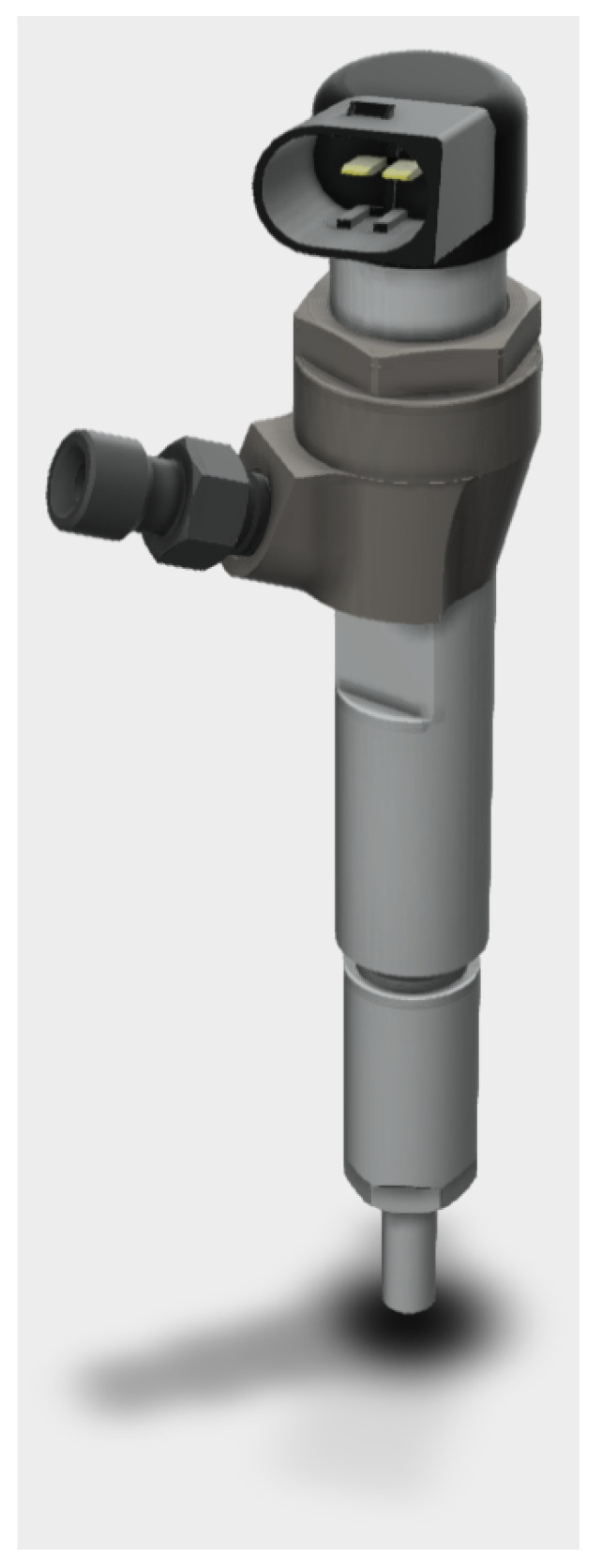
HPCR solenoid injector.

Figure 3 draws a general layout schema of an HPCR system. It mainly consists of a pipe with fixing flanges. Internal rail volume is accessible through a tube to the high-pressure pump and pressure lines connecting the injectors in parallel. To obtain the desired injection pressure, both injection starting time and its duration must be electrically actuated by triggers released by the ECU. An example of a solenoid injector is reported in Figure 4. It can be observed the electric contacts for the solenoid coil (top), which receive triggers from the ECU, and the high-pressure connector (middle), which joins the injector to the HPCR system.

**Figure 5:**
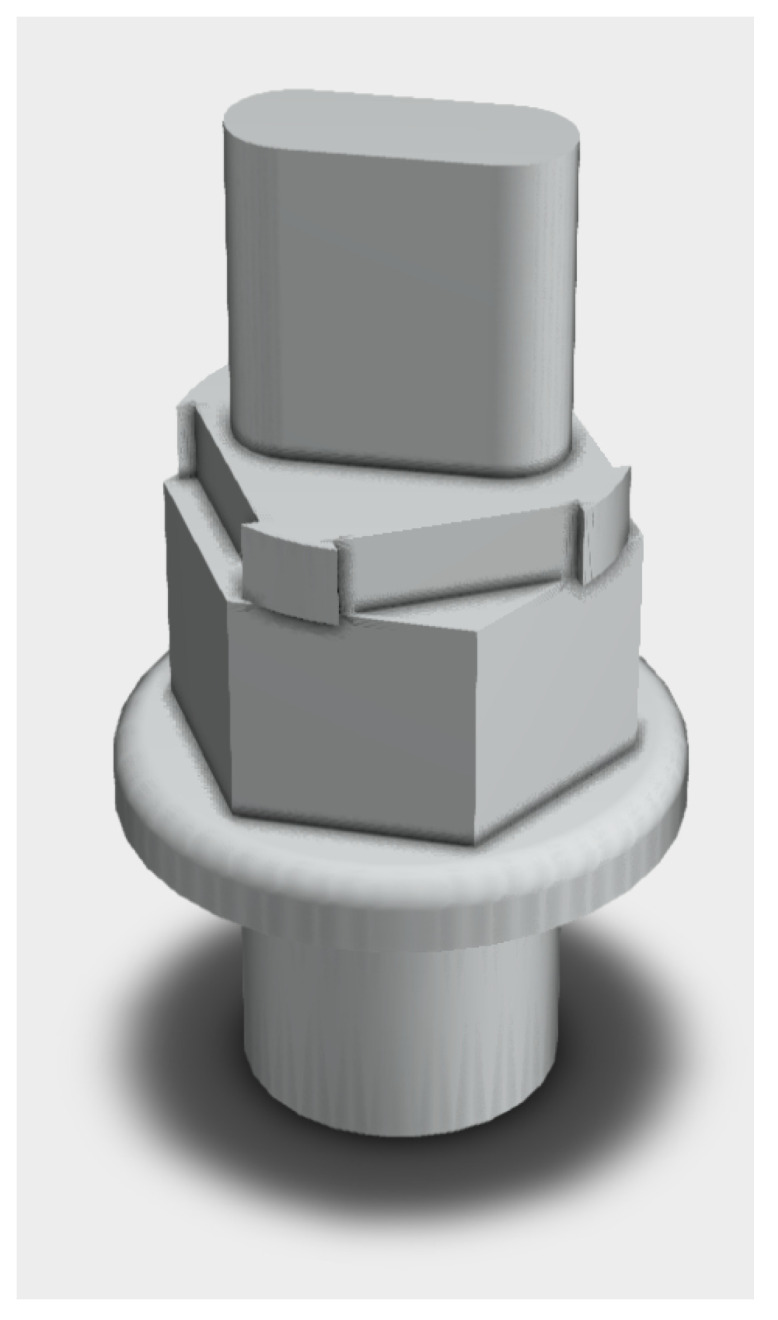
HPCR pressure valve.

In an HPCR system, it is essential to maintain the stability of the injection pressure and reduce the difference in the fuel-injection amount caused by different injectors in the system. Compression and rarefaction waves inside the rail may be caused by suddenly fuel acceleration and deceleration with the result of degrading the injection precision [38]. Under certain load conditions, a fault may also occur through a flash opening of the injector causes an unspecified pressure drop in the rail. The diagnostic system reads these events as the repeated opening of a worn-out pressure relief valve (Pressure Relief Valve—PRV). A PRV for an HPCR system incorporates a ceramic spherical valve element that moves into and out of contact with a conical valve seat of a metallic valve body. This safety component is equipped on some HPCR systems, particularly heavy-duty diesel engines, to prevent high pressures in the rail and fuel to flow back. This avoids a potentially dangerous condition for the diesel engine [18]. An example of an HPCR pressure valve system is depicted in Figure 5.

### 6.1. Multisensor Dataset

To get a deeper insight into the targeted fault and to explain the causes of the anomalous injection events, experiments were carried out on a six-cylinder, four-stroke, turbocharged, heavy-duty diesel engine equipped with an HPCR fuel-injection system. The injection system is constituted of one high-pressure fuel pump, one common-rail pipe, and six injectors. The high-pressure fuel is delivered from the pump to the common-rail and finally to the injector in each cylinder of the engine.

The acquisition of process data was made possible by a memory emulator module associated with the ECU employed to increase its storage capacity. The total number of sensors is 34. The list of sensors is reported in Table 1.

Collected measurements were recorded by sensors placed on the vehicle, while a few of them were related to actuator signals generated by the ECU during the injection process. Only two channels were related to the on-board diagnostic system of the PRV (labels 33 and 34). Channels were related to all principal variables related to the HPCR injection process. In our study, data measured in the post-treatment system were not considered because, given the latency of the exhaust flow, these variables resulted shifted in time from the instant of the detected fault event.

The targeted fault is an unusual opening of the injector, which leaks a quantity of fuel that causes pressure drops in the rail. When these fluctuations are significant, the diagnostic system interprets the phenomenon inaccurately. On-board diagnostic releases an alarm concerning the opening of the PRV. Although it allows the fuel backflow only in situations of pressure overshoot, PRV is a passive safety component, implying that it is neither equipped with sensors nor can be controlled for opening. Any wear resulting in leakage is evaluated indirectly through a deviation of the rail pressure signal. When the magnitude of the fault is not sufficient to trigger the warning, it is difficult to discriminate this event from regular injections. Both scenarios could lead to a similar rate of rail pressure reduction. Therefore, evaluating only rail pressure profile variability is ineffective. To understand the progression of the injection process before and after fault events, it is important to evaluate all the sensor measurements.

**Figure 6:**
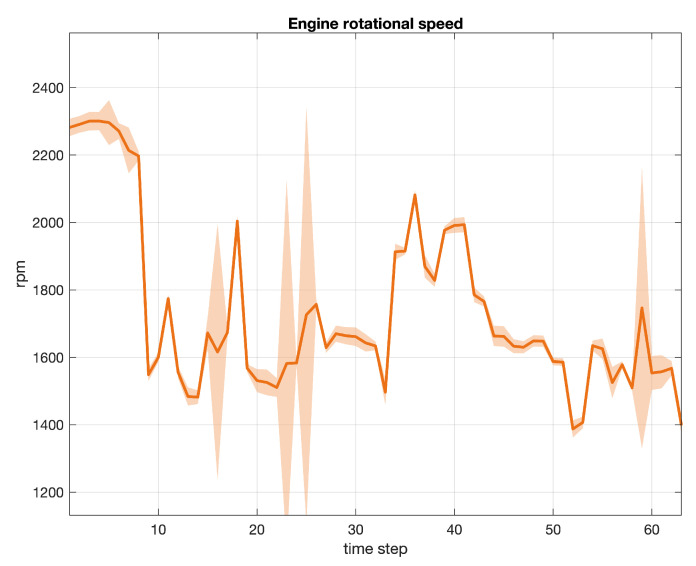
Engine rotational speed (label 1). Average value (continued bold line) and 3-sigma area plot for 203 samples.

**Figure 7:**
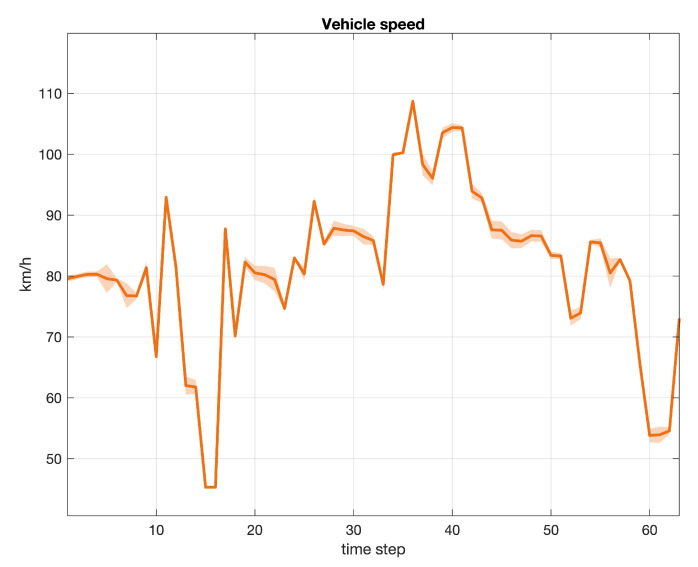
Vehicle speed (label 2). Average value (continued bold line) and 3-sigma area plot for 203 samples.

**Figure 8:**
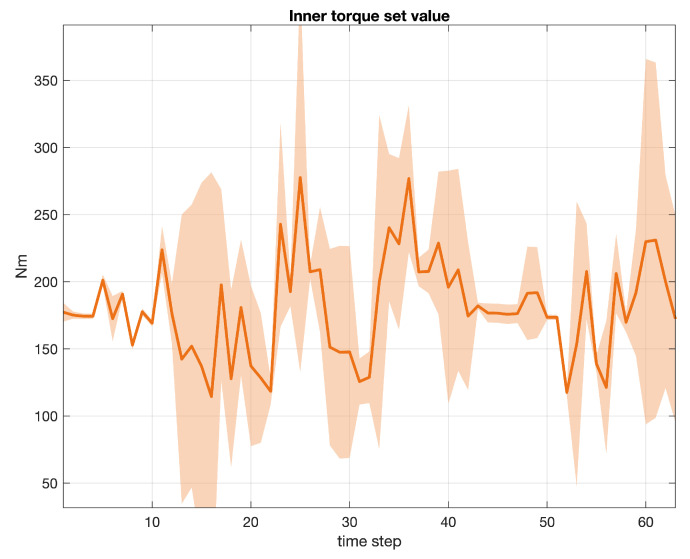
Inner torque set value (label 12). Average value (continued bold line) and 3-sigma area plot for 203 samples.

Three examples of signals collected during our experiments are depicted in subsequent Figure 6, Figure 7 and Figure 8. In particular, each figure describes the average value of the signal, with a continued bold line, including the area of variability computed as the 3-sigma interval from the mean value for each time step. The total number of samples considered in each graph is equal to 203. Figure 6 refers to the engine rotational speed (label 1 in Table 1), Figure 7 to the vehicle speed (label 2 in Table 1), while Figure 8 to the inner torque set value (label 12 in Table 1). Every signal recorded by a sensor in a given time window was linearly scaled in the range [0,1] and centered by subtracting the average profile of the relative variable.

The final dataset for fault diagnosis was obtained by considering the series of Diagnostic Trouble Codes (DTCs) triggered by the ECU corresponding to the opening of the PRV. For each scaled and centered signal associated with a specific sensor, the values related to instants of DTCs produced by the ECU were collected. The total number of such events in the monitored window (number of DTCs released by the ECU) was equal to 1101. Therefore, these operations produced a dataset on the form of a matrix $X_{c,[0,1]}\in R^{P\times M}$ with M=1101 observations and P=34 process measurements, subsequently processed by PCA to extract relevant features and to reduce the dimensionality of multisensor process data. Specifically, 7 PCs were derived from the original dataset of data from 34 sensors. To extract the feature and to choose the number of PCs, conventional cross-validation statistical techniques were implemented on data [39]. Such N=7 PCs correspond to about 90% of explained variability in the data, while the first 4 PCs only correspond to about 85% of explained variability in the data.

### 6.2. Clustering

Cluster analysis aims to arrange observations considered similar to reveal patterns that support the investigation on the targeted fault (the pressure drop in the injection process). This assists the practitioner in the root-cause analysis by highlighting the set of components to which the fault can be ascribed.

Three clustering methods in our case study: (i) the *K*-means++, (ii) the original NJW SC, and (iii) the proposed NJW SC with pairwise constraints. The three methods were applied to the dataset of N=7 scores obtained from PCA.

**Figure 9:**
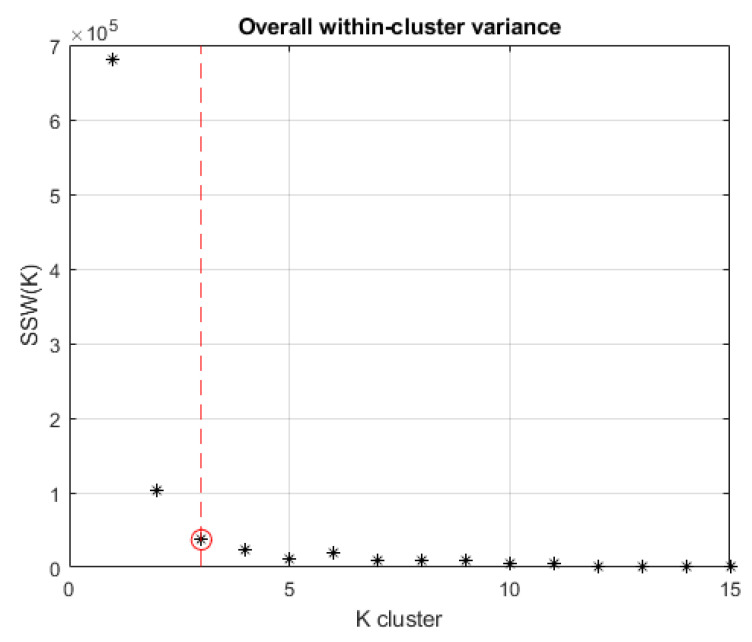
Kneedle algorithm results applied to *K*-means++ method.

**Figure 10:**
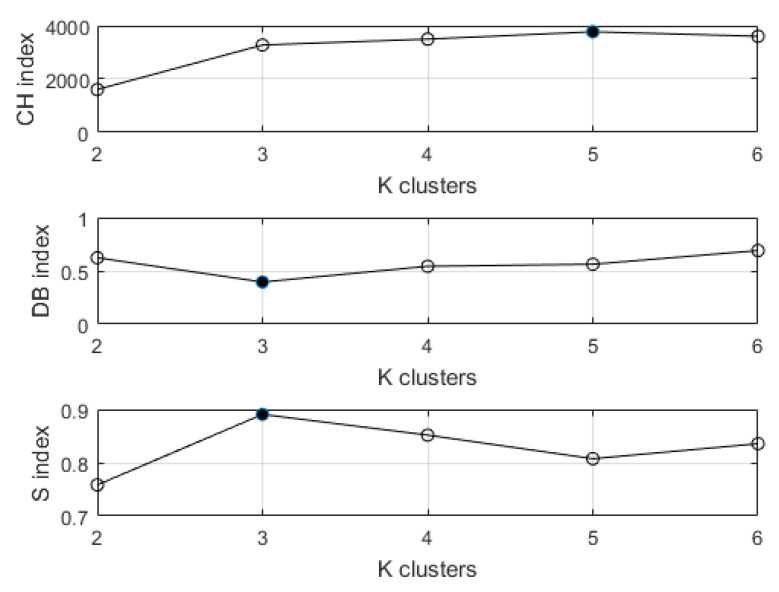
Internal validation indexes of the performed clustering.

In *K*-means++ clustering, defining the optimal number of clusters was handled by *Kneedle*. It consists of minimizing the intra-cluster variance of SSW. The results of the *Kneedle* algorithm applied to the case study dataset are depicted in Figure 9. The resulting number of optimal clusters is equal to K=3. To validate the classification results, the Caliński-Harabasz (CH in Equation (3)), Davies-Bouldin (DB in Equation (4)), and Silhouette (*S* in Equation (5)) indices were computed. Figure 10 shows that the value of K=3 appears to be optimal according to DB and *S* indexes, although the CH exhibits a slightly greater value for K=5 clusters.

**Figure 11:**
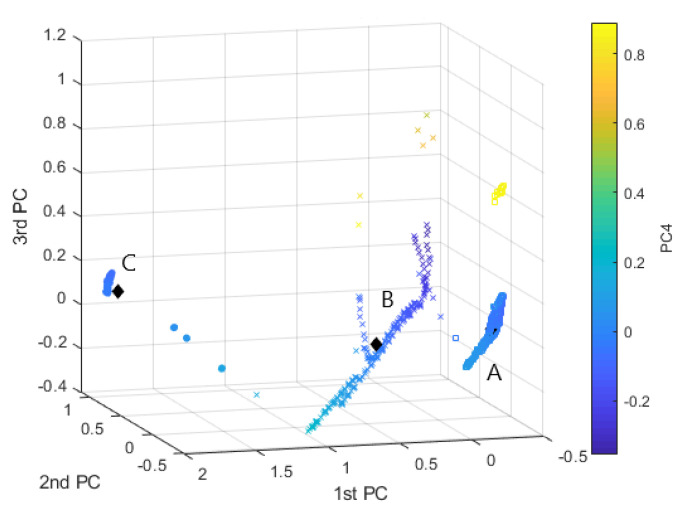
*K*-means, K=3. Clustering representation in the principal component scatterplot, Clust.A—“□”, Clust.B—“*x*”, Clust.C—“∘”.

Figure 11 displays the K=3 clusters obtained by *K*-means++, in a three-axis diagram, where each axis is related to the first 3 PC. A color scale is used to express the value of the 4th PC (the first 4 PCs describe 85% of variability). From Figure 11 it can be noticed that several outliers (points distant from the relative centroid of the cluster) are assigned to clusters A (square graphical symbol) and B (x graphical symbol) and are characterized by a high value of the 4th PC.

**Figure 12:**
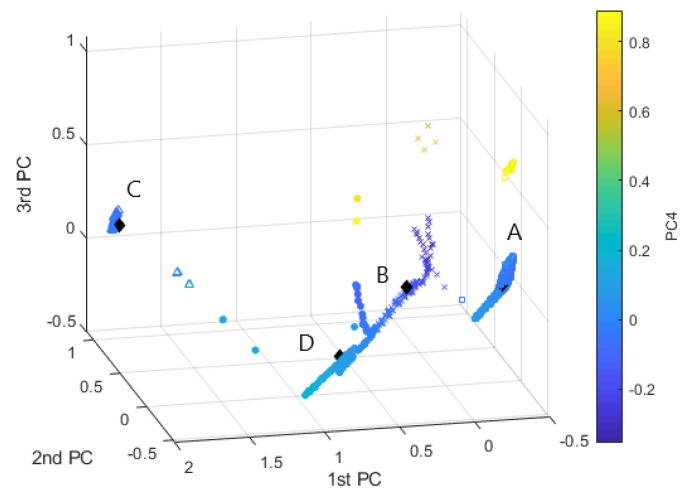
SC method. Clustering representation in the principal components scatterplot: Clust.A—“□”, Clust.B—“*x*”, Clust.C—“△”, Clust.D—“∘”.

We implemented the SC method on the same multisensor dataset with $L_{\mathrm{sym}}$ and constructed the spectral embedding according to the NJW algorithm [8]. The eigenvalues of $L_{\mathrm{sym}}$ were estimated as the largest empirical eigengap $\hat{K}={arg max}_{i}\lambda_{i+1}-\lambda_{i}$, which resulted in the value $\hat{K}=4$. By applying SC to all scores, it is possible to determine the classification as in Figure 12. It can be observed that SC results in splitting one of the clusters into two different partitions.

**Figure 13:**
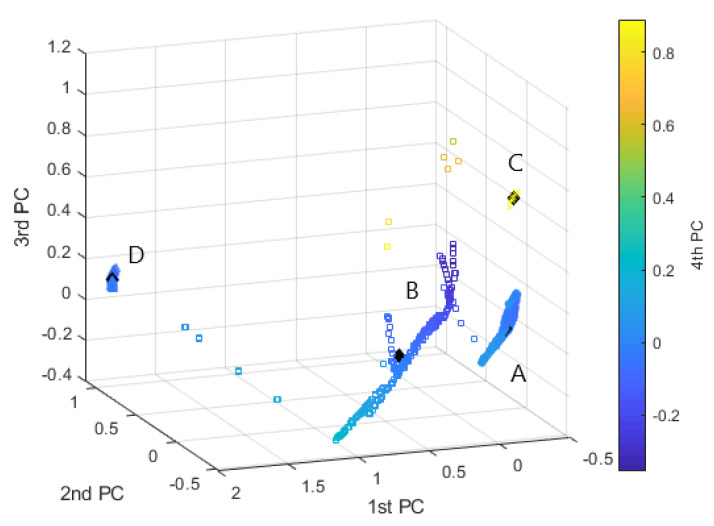
SC method with pairwise constrains. Clustering representation in the principal components scatterplot: Clust.A—“∘”, Clust.B—“□”, Clust.C—“*x*”, Clust.D—“△” (SC, K=4).

Ultimately, we implemented the SC method by adding 7 must-link constraints and 8 cannot-link constraints, which were obtained from experienced personnel for 15 specific events out of the 1101 faults collected, which represents a small fraction of the dataset. From the results graphically depicted in Figure 13 it can be observed that the SC approach augmented with pairwise constrains can accurately partition the set of data by isolating the points characterized by a high value of the 4th PC and that is distantly positioned from the centroid of the dense clusters.

To confirm the results obtained by the original SC NJW algorithm and the SC NJW augmented with pairwise constraints, the validation indexes were computed and are reported in Table 2. Bold font represents outperforming results. It can be noted that while CH presents a decreasing performance level of around 2%, the DB and *S* indexes show an improved performance of the SC NJW augmented with pairwise constraints of about 16% and 4% when compared to the original SC.

### 6.3. Cluster Evaluation of Fault Scenario

To evaluate the content of a cluster and to label the classes they represent, the parallel coordinate plot [40] of cluster centroid of interest was examined. The cluster centroid is computed as the barycentre of a discrete points distribution and hence of the cluster. In this regard, a centroid can be considered the most representative point of a cluster if that is compact and dense, as for clusters *A* and *C* obtained by SC (Figure 13).

Both clusters *A* and *C* describe conditions under which the torque (label 4 in Table 1) of the engine is maximum, as well as all related profiles (injected quantity of fuel); for this reason, clusters *A* and *C* are labeled *“Full load”*. The comparison, obtained by superimposing the combination of centroid coordinates, allows us to emphasize the fault, the operating conditions related to this fault event, and above all, the progression of the process.

**Figure 14:**
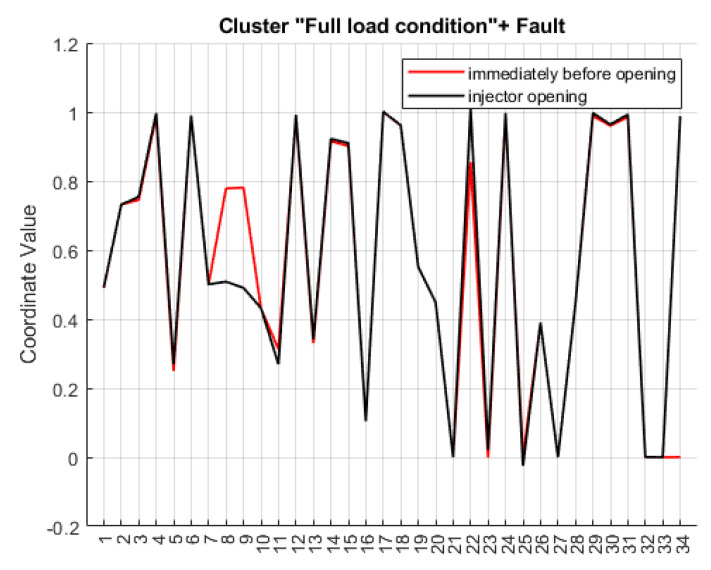
Comparison between cluster *A* (red) and cluster *C* (black) centroid coordinates. SC with pairwise constrains.

The analysis of Figure 14 reveals that the coordinates of centroids are similar except for the variables with labels No. 8 and No. 9 related to the rail pressure gradient (Table 1). The pressure drop, and therefore the fault, is imputable to a malfunction of the injector.

From Figure 14, it is clear that the scenario demonstrated by the two clusters is the same and matches the condition of maximum torque demand. Activation of the last variable No. 34 (in cluster *C*) is associated with a reduction in the pressure gradient monitored by variables No. 7 and No. 8. Furthermore, differences are present in channel No. 22, the energizing time of the injector: this is attributable to the dependence of this variable on the rail pressure, which is not capable of following the set point. This gap is also shown by the slight differences between the values of channel No. 10 and No. 11.

As a result, cluster *C* presents the specific problem of the common rail system under investigation, namely the pressure loss of the fuel that is not attributable to normal operation. In the extracted pattern, the filtered pressure is lower than the set pressure at a consistent rate. Since the rail pressure regulation is in a closed loop, the pressure drop event connected to the injector fault occurs so rapidly that the system does not compensate for the deviation immediately. Variable No. 22 represents the energizing time during the main injection, and it can be noticed that it increases in the fault scenario. The pressure drop, and therefore the fault, is clearly due to a malfunction of the injector.

## 7. Conclusions

Clustering algorithms, which group similar features into the same cluster and separate dissimilar features into different ones, are common analysis methods for unlabeled data. The clustering phase is an essential aspect of the analysis of multisensor data. In this study, to have a better insight of the faults in the injection process, clustering has been applied to multisensor measurements obtained by experiments on a real diesel engine, equipped with an HPCR system and electronically controlled by an ECU. Clustering, exploiting the compactness of the space constituted, has contributed to identifying different scenarios allowing us to diagnose the root causes of the targeted fault in different operating areas of the engine.

The most widely used clustering algorithm, *K*-means, although distinguishing such zones, has failed to identify a fault scenario. Using this classification, clusters are misconstrued because the *K*-means is sensitive to non-spherical structures of data, altering the cluster centroid computation, which supports fault diagnosis. In the case study presented in this paper, SC provides the advantage of an aggregation criterion more robust to non-spherical structures of data. In this paper, a semi-supervised approach has also been discussed to combine labeled and unlabeled measurement data in SC modeling. A class of fault has been identified within the resulting groups of clusters, contributing to a comprehensive understanding of the phenomenon.

In this study, the PCA was implemented as a dimensional reduction procedure to improve clustering by decreasing the dimension of the measurement dataset while capturing the linear correlation structure of the data. The PCA replaces the measurements with a smaller number of points that are a linear combination of original data and considers these new points as the scalar variables. However, this approach may mask the effect of each sensor by merging them into new ones and fails to exploit the ordering structure of a variable. In the situations in which many of the inputs are not informative, the extracted features may become diluted. A direction of future research includes replacing the PCA step with a variable screening phase for fault diagnosis. This method should perform variable selection to distinguish which inputs are most informative in the original measurement domain, i.e., a method capable of sensor screening by selecting the most informative variable inputs. A recent example in the literature of a statistical method to perform sensor screening, and to generate predictions, is reported in [41] concerning a case study related to the monitoring of an internal combustion engine through a large number of sensor signals.

Considering the experimental approach of this work, clustering techniques are showed to be the main tool for assisting fault diagnosis in modern applications. Facilitating fault examination procedures would contribute to improving root-cause analysis, bypassing the need for a deep knowledge of the process, and so for the support of experienced personnel.

## Figures and Tables

**Table 1:** List of channels. For each channel is reported the description, unit of measure and processing label.

Channel Name	Unit of Measure	Label
Average engine speed	[rpm]	1
Vehicle speed	[km/h]	2
Air mass flow	[kg/h]	3
Current injection quantity	[mg/hub]	4
Metering Unit actual current	[mA]	5
Battery voltage	[mV]	6
Downstream engine coolant temperature	[${}^{\circ }$C]	7
Measured rail pressure gradients (new)	[hPa]	8
Measured rail pressure gradients (old)	[hPa]	9
Rail pressure set point	[hPa]	10
Rail pressure filtered	[hPa]	11
Inner torque set value	[Nm]	12
Set point volume flow of RP governing	[mm^3^/s]	13
Volume flow requested overall for all injection system	[mm^3^/s]	14
Volume flow requested for all injections	[mm^3^/s]	15
1st–6th Cylinder individual correction quantity	[mg/hub]	16–21
Injection time (main)	[μs]	22
Injection time (pilot)	[μs]	23
Desired main injection quantity	[mg/hub]	24
Desired pilot injection quantity	[mg/hub]	25
Quantity correction for limiting pressure variations	[mg/hub]	26
Actual fuel supply low pressure	[bar]	27
Value of fuel temperature	[${}^{\circ }$C]	28
Sum of requested injection quantity per cyl.	[mg/hub]	29
Sum of all estimated injection control quantities	[mg/hub]	30
Torque generating engine fuel-injection quantity	[mg/hub]	31
Status of fuel balancing control loop	[-]	32
State variable of PRV	[-]	33
PRV opening detection	[-]	34

**Table 2:** Internal validation measures for spectral clustering.

Index	Original SC	SC with Pairwise Constraints
CH	**2495**	2438.3
DB	0.4162	**0.3481**
S	0.8528	**0.8840**
